# Complete Genome Sequence of a Clinical Isolate of Acinetobacter baumannii Harboring 11 Plasmids

**DOI:** 10.1128/MRA.00695-21

**Published:** 2021-09-30

**Authors:** Arata Sakiyama, Yuki Matsumoto, Taishi Tsubouchi, Masato Suzuki, Makoto Niki, Mamiko Niki, Ken-Ich Oinuma, Hiroshi Kakeya, Yukihiro Kaneko

**Affiliations:** a Department of Bacteriology, Graduate School of Medicine, Osaka City University, Abeno, Osaka, Japan; b Research Center for Infectious Disease Sciences, Graduate School of Medicine, Osaka City University, Abeno, Osaka, Japan; c Toneyama Institute for Tuberculosis Research, Graduate School of Medicine, Osaka City University, Toneyama, Osaka, Japan; d Antimicrobial Resistance Research Center, National Institute of Infectious Diseases, Aoba, Higashi-Murayama, Tokyo, Japan; e Department of Infection Control and Prevention, Osaka City University Hospital, Abeno, Osaka, Japan; University of Maryland School of Medicine

## Abstract

Here, we report the complete genome sequence of an Acinetobacter baumannii isolate harboring 11 plasmids, obtained at a hospital in Japan in 2016. The complete 4.07-Mbp genome sequence (1 chromosome and 11 plasmids) was analyzed by a combination of long-read (Flongle) and short-read (NovaSeq 6000) sequencing.

## ANNOUNCEMENT

Acinetobacter spp. are naturally competent at DNA uptake and transformation using type IV pili (1), contributing to the horizontal gene transfer (HGT) of antimicrobial resistance genes and virulence genes. We isolated an Acinetobacter sp. strain, obtained from the venous blood of a patient in Japan in 2016 (IRB approval number 3568; 30 September 2016). When we extracted and electrophoresed the genomic DNA (gDNA), we found that the strain had multiple plasmids. We thought that sequencing the genome of this strain would help elucidate the plasmid acquisition mechanism of Acinetobacter species.

The blood culture became positive at 21.9 h, and the bacteria were then isolated on sheep blood agar and MacConkey agar solid medium at 35°C for 24 h. The isolates were analyzed using a MicroScan WalkAway 40 system equipped with a Neg NF combo panel (Beckman Coulter, Brea, CA, USA) and determined to be an Acinetobacter sp.

OCU_Ac18 was streaked onto a Mueller-Hinton II agar plate and incubated at 37°C for 16 h. gDNA was extracted using the DNeasy blood and tissue kit (Qiagen). Whole-genome sequencing was performed using the MinION system with FLO-FLG001 (Oxford Nanopore Technologies [ONT]) and the NovaSeq 6000 system (Illumina). The gDNA was sheared to 8 kbp using a g-TUBE device (M&S Instruments) and prepared using a ligation sequencing kit (SQK-LSK108) for the ONT library. The Illumina library (paired-end format; insert size, 500 to 900 bp) was prepared using the Nextera XT DNA library prep kit. The ONT reads were base called using Guppy v3.2.1 in high-accuracy mode. The read *N*_50_ value and the number of reads were 6.92 kbp and 87,219, respectively. The ONT reads were filtered using Filtlong v0.2.0 (https://github.com/rrwick/Filtlong; length, ≧1,000) to keep only 90% of reads, with a target output of 500 Mb (*N*_50_, 7.08 kbp). The Illumina reads (read length, 151 bp; number of reads, 31,907,116) were filtered using Fastp v0.20.1 to a quality score of ≧30, trimming 5 bases off the 3′ end (2). Both sets of filtered reads were assembled *de novo* using Unicycler v0.4.8 (3). The assembly yielded 12 circular contigs, and the *dnaA* gene and *rep*-like gene on OCUAc18-4 and pOCUAc18-6 were rotated to start at the first gene. The *rep*-like genes of other contigs were annotated using the RAST v2.0 server (4) and were rotated manually to put the *rep*-like gene first. Sequencing errors such as indels were corrected four times using Pilon v1.24 (5). The complete genome sequence of OCU_Ac18 was constructed as one chromosome and 11 plasmids (Table 1). A total of 3,863 coding DNA sequences (CDSs) were annotated using the DDBJ Fast Annotation and Submission Tool (DFAST) server (6). The complete genome sequence of OCU_Ac18 was compared to the complete genome sequence of A. baumannii ATCC 19606^T^ (GenBank accession number AP022839) using average nucleotide identity (ANI) methods, calculated using pyani v0.2.10. The ANI between the genomes was 97.50%, so we finally identified OCU_Ac18 as A. baumannii.

### Data availability.

The complete genome sequence of OCU_Ac18 has been deposited in DDBJ/ENA/GenBank under the accession numbers described in Table 1, and the raw sequence data are available in the Sequence Read Archive under the accession numbers DRX291375 and DRX291376.

**TABLE 1:** Sequencing metrics for the OCU_Ac18 genome

Genome element	Sequence length (bp)	G+C content (%)	No. of reads	DDBJ accession no.
OCU_Ac18chr	3,920,683	39.2	24,992,837	AP024802
pOCUAc18-1	90,090	40.4	594,136	AP024803
pOCUAc18-2	12,817	35.9	604,436	AP024804
pOCUAc18-3	10,831	35.8	735,450	AP024805
pOCUAc18-4	8,948	34.8	471,670	AP024806
pOCUAc18-5	7,104	37.8	516,595	AP024807
pOCUAc18-6	5,049	37.2	626,625	AP024808
pOCUAc18-7	4,293	41.1	476,726	AP024809
pOCUAc18-8	3,948	41.0	412,868	AP024810
pOCUAc18-9	3,496	36.8	390,525	AP024811
pOCUAc18-10	2,444	36.4	407,282	AP024812
pOCUAc18-11	2,278	39.9	436,954	AP024813
